# Supplementary material for: Liquid water on cold exo-Earths via basal melting of ice sheets
Source: Nat Commun. 2022 Dec 6;13:7521. doi: 10.1038/s41467-022-35187-4 (PMC9726705; doi:10.1038/s41467-022-35187-4)
Supplement: Supplementary file 1 — Supplementary Information [file 41467_2022_35187_MOESM1_ESM.pdf]

Supplementary Information for

Liquid Water on Cold Exo-Earths via Basal Melting of Ice Sheets

Lujendra Ojha[1], Bryce Troncone[1], Jacob Buffo[2], Baptiste Journaux[3], George McDonald[4]

[1]Department of Earth and Planetary Sciences, Rutgers University, Piscataway, NJ, USA
[2]Thayer School of Engineering, Dartmouth College, Hanover, NH, USA.
[3]Department of Earth and Space Science, University of Washington, Seattle, WA, USA.
[4]Department of Earth Sciences, University of Oregon, Eugene, OR, USA.

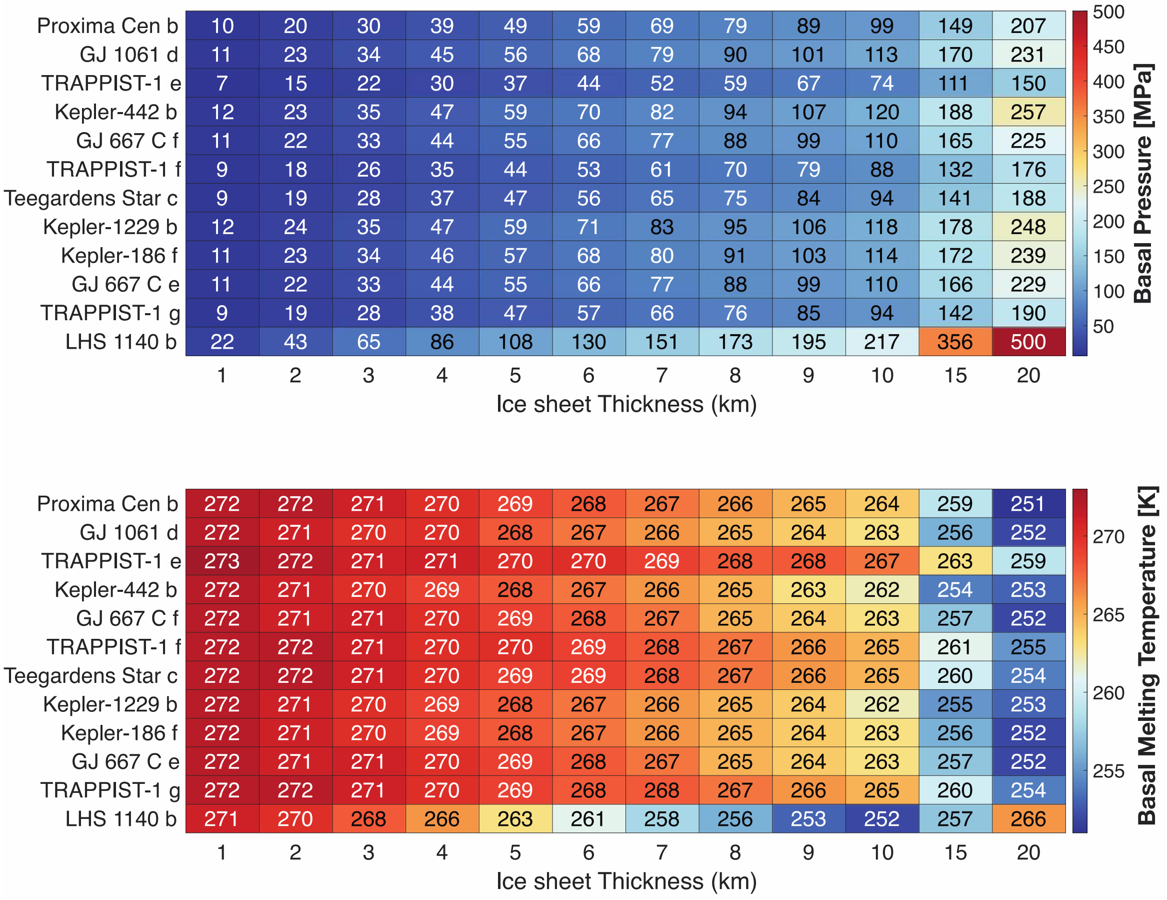

Supplementary Figure 1. Pressure (top) and melting temperature (bottom) at the base of ice sheets of various thicknesses on exo-Earths. The pressure at the bottom of the ice sheet increases with depth, but the melting temperature of ice initially decreases due to the presence of pressurized ice Ih. However, if the basal pressure exceeds 200 MPa, as would be the case for thick ice sheets on LHS 1140 b, the melting temperature increases due to the presence of dense high-pressure ice polymorphs.

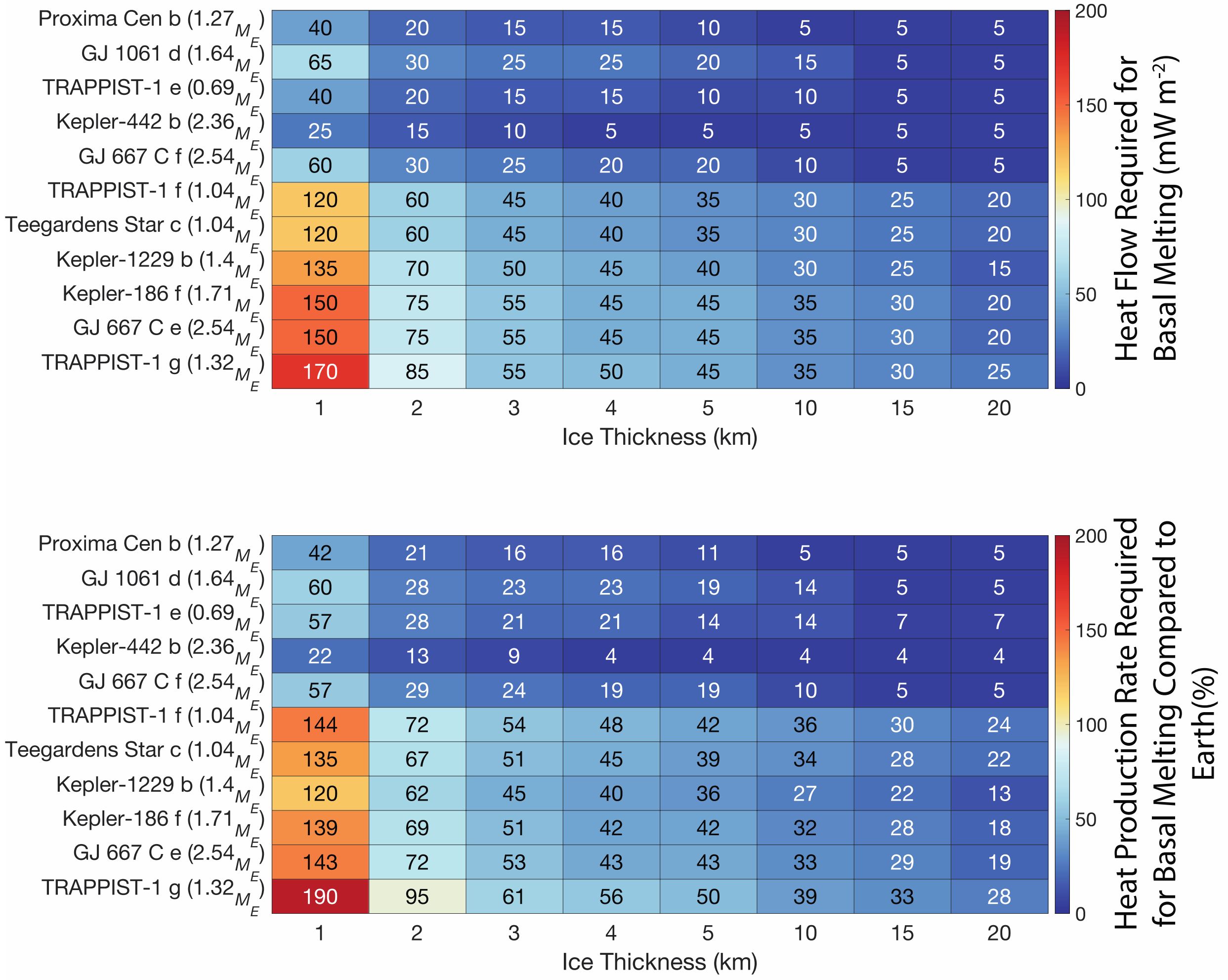

Supplementary Figure 2. Heat flow (top) and heat production rate per unit mass (bottom) required for basal melting on various exo-Earths as a function of the ice sheet thickness. Heatmap showing the average heat flow required for basal melting on various exo-Earths. See Table 1 for the surface gravity, global equivalent layer (GEL) of water assuming Earth-like WMF, and T_s estimate of these bodies. Heat production rate per unit mass, relative to Earth, required for basal melting on various exo-Earths as a function of the ice sheet thickness. The change in the text color is only for clarity.

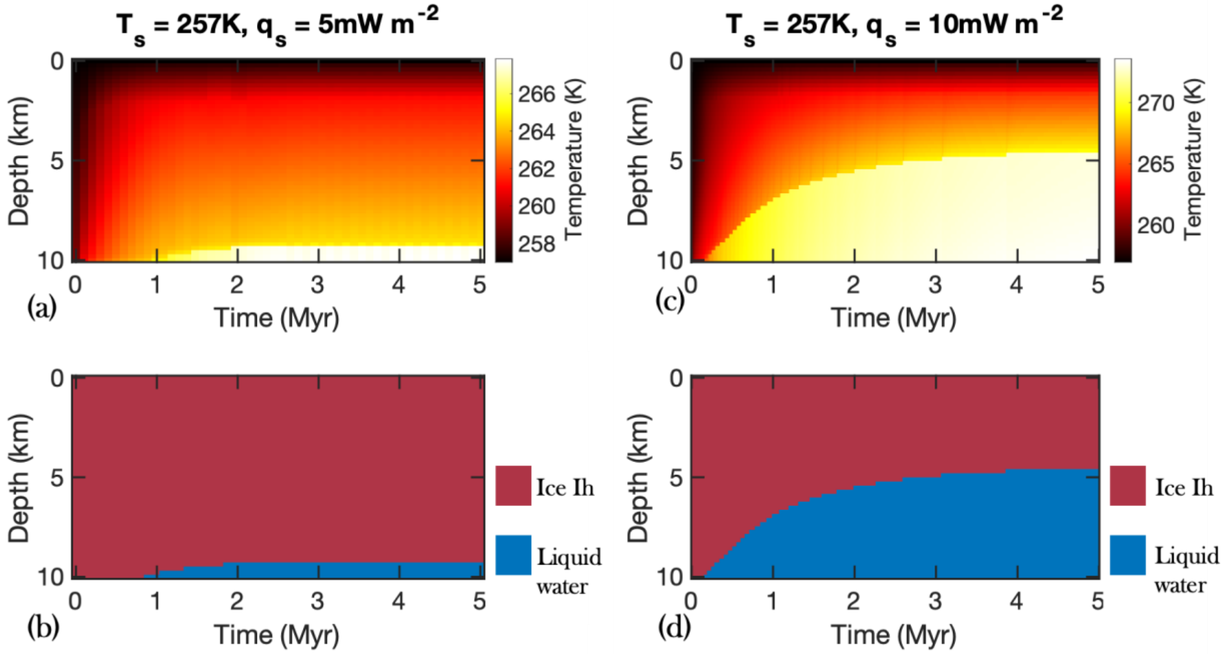

Supplementary Figure 3. Temperature and ice phases as a function of depth within a 10 km thick ice sheet on Proxima Centauri B. **(a)** Temperature distribution as a function of depth and time on Proxima Centauri B for a 10 km thick ice sheet, T_s of 257 K, and heat flow of 5 mW m^{-2}. **(b)** Ice phase evolution as a function of depth and time on Proxima centauri B over a 5-million-year time period. (c) same as (a) but assuming heat flow of 10 mW m^{-2}. (d) Same as (b), but assuming heat flow of 10 mW m^{-2}. The thickness of the basal melt significantly increases with higher heat flow.

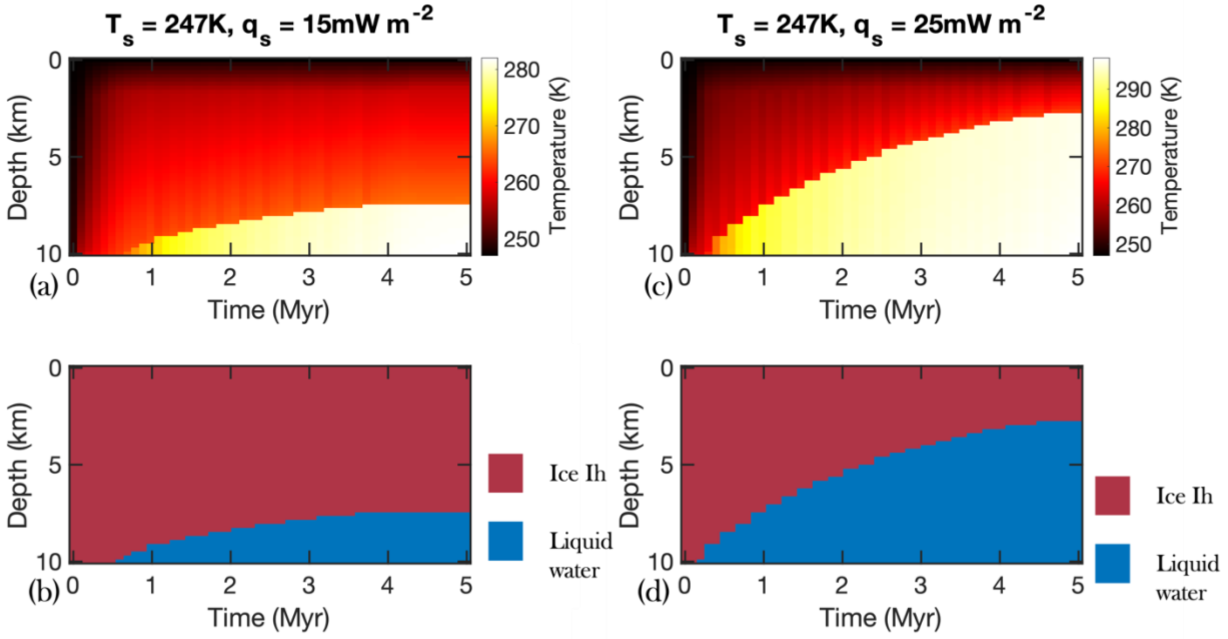

Supplementary Figure 4. Temperature and ice phases as a function of depth within a 10 km thick ice sheet on GJ 1061 d. **(a)** Temperature distribution as a function of depth and time on GJ 1061 d for a 10 km thick ice sheet, T_s of 247 K, and heat flow of 15 mW m^{-2}. **(b)** Ice phase evolution as a function of depth and time on GJ 1061 d over a 5-million-year time period. (c) same as (a) but assuming heat flow of 25 mW m^{-2}. (d) Same as (b), but assuming heat flow of 25 mW m^{-2}. The thickness of the basal melt significantly increases with higher heat flow.

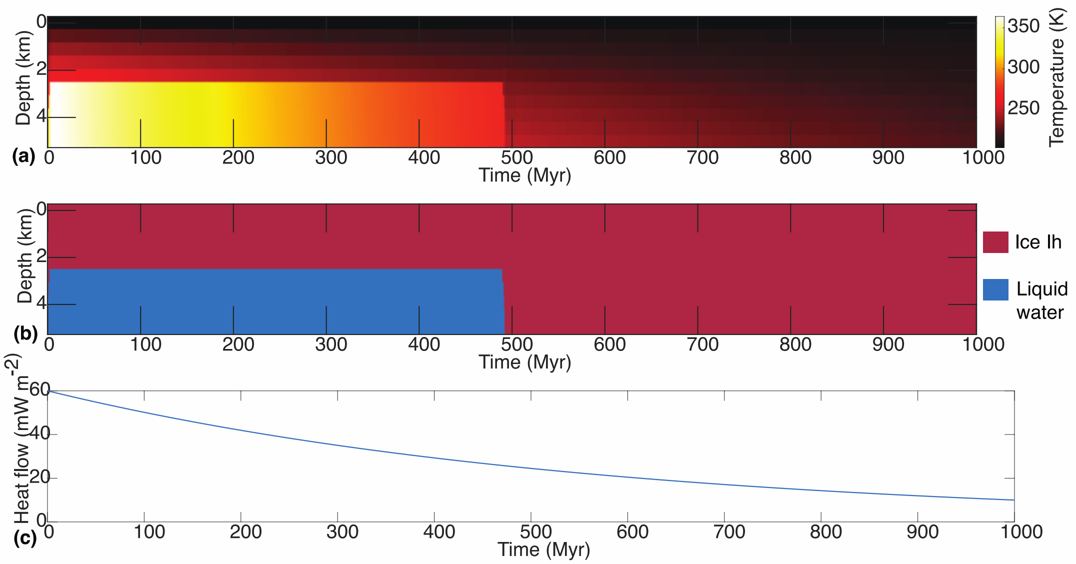

Supplementary Figure 5. Temperature and ice phases as a function of depth within a 5-km thick ice sheet on TRAPPIST-1 g, assuming exponentially declining heat flow over a 1-billion year period. **(a)** Temperature distribution as a function of depth and time on Proxima Centauri B for a 1 km thick ice sheet assuming T_s of 257 K and variable heat flow over a billion years. **(b)** Ice phase evolution as a function of depth and time on Proxima Centauri B over a billion years. **(c)** An ad-hoc exponential function that models heat loss on Proxima Centauri B from 60 mW m^{-2} to 30 mW m^{-2} over a billion years.

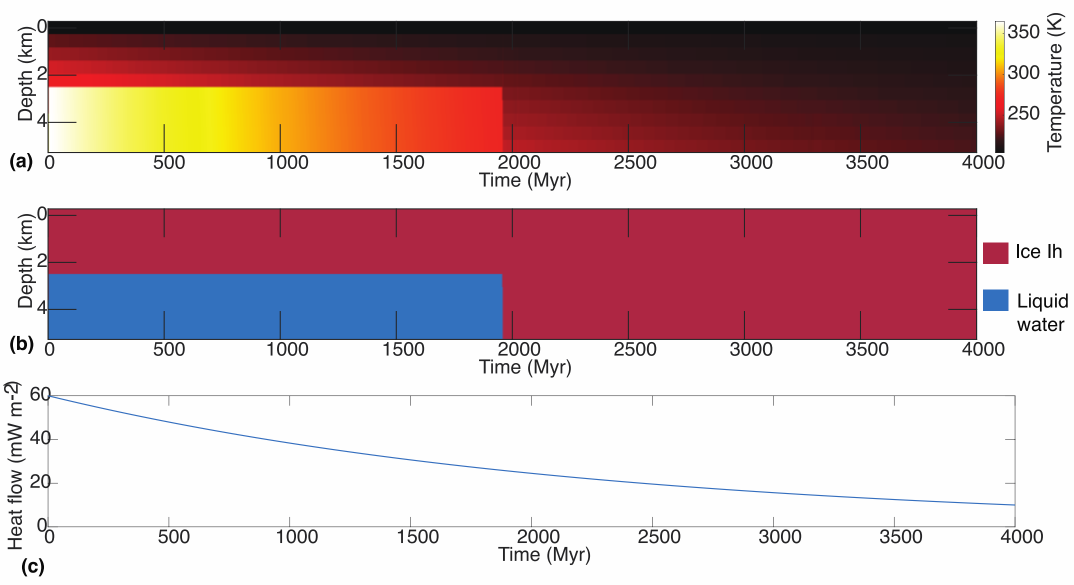

Supplementary Figure 6. Temperature and ice phases as a function of depth within a 5-km thick ice sheet on TRAPPIST-1 g, assuming exponentially declining heat flow over a41-billion year period. **(a)** Temperature distribution as a function of depth and time on Proxima Centauri B for a 1 km thick ice sheet assuming T_s of 257 K and variable heat flow over 4 billion years. **(b)** Ice phase evolution as a function of depth and time on Proxima Centauri B over 4 billion years. **(c)** An ad-hoc exponential function that models heat loss on Proxima Centauri B from 60 mW m^{-2} to 30 mW m^{-2} over 4 billion years.

Phase	η_0 (Pa s)	K_{avg} (W m^{-1} K^{-1})
Liquid water	1.8×10^{-3}	0.6
Ice Ih	5×10^{13}	2.4
Ice II	10^{15}	1.8
Ice III	10^{12}	1.1
Ice V	10^{16}	1.5
Ice VI	10^{14}	1.9

Supplementary Table 1. *Characteristic viscosities of planetary ices used during our investigation. Values from [1-3]. Average thermal conductivity used in our investigation of the thermophysical evolution of ice on exo-Earths [4].*

References

1. Echelmeyer, K. and B. Kamb, *Rheology of ice II and ice III from high-pressure extrusion.* Geophysical Research Letters, 1986. **13**(7): p. 693-696.
2. Poirier, J., C. Sotin, and J. Peyronneau, *Viscosity of high-pressure ice VI and evolution and dynamics of Ganymede.* Nature, 1981. **292**(5820): p. 225-227.
3. Sotin, C. and J. Poirier, *Viscosity of ice V.* Le Journal de Physique Colloques, 1987. **48**(C1): p. C1-233-C1-238.
4. Andersson, O. and A. Inaba, *Thermal conductivity of crystalline and amorphous ices and its implications on amorphization and glassy water.* Physical Chemistry Chemical Physics, 2005. **7**(7): p. 1441-1449.
